# An Introduction to the Main Types of Economic Evaluations Used for Informing Priority Setting and Resource Allocation in Healthcare: Key Features, Uses, and Limitations

**DOI:** 10.3389/fpubh.2021.722927

**Published:** 2021-08-25

**Authors:** Hugo C. Turner, Rachel A. Archer, Laura E. Downey, Wanrudee Isaranuwatchai, Kalipso Chalkidou, Mark Jit, Yot Teerawattananon

**Affiliations:** ^1^MRC Centre for Global Infectious Disease Analysis, School of Public Health, Imperial College London, London, United Kingdom; ^2^Health Intervention and Technology Assessment Program, Ministry of Public Health, Nonthaburi, Thailand; ^3^School of Public Health, Imperial College London, London, United Kingdom; ^4^Institute of Health Policy, Management and Evaluation, University of Toronto, Toronto, ON, Canada; ^5^Department of Infectious Disease Epidemiology, Faculty of Epidemiology and Population Health, London School of Hygiene and Tropical Medicine, London, United Kingdom; ^6^Modelling and Economics Unit, Public Health England, London, United Kingdom; ^7^Saw Swee Hock School of Public Health, National University of Singapore, Singapore, Singapore

**Keywords:** economic evaluations, cost-effectiveness (economics), cost-utility analysis, cost-benefit analysis, cost-effectiveness analyses

## Abstract

Economic evidence is increasingly being used for informing health policies. However, the underlining principles of health economic analyses are not always fully understood by non-health economists, and inappropriate types of analyses, as well as inconsistent methodologies, may be being used for informing health policy decisions. In addition, there is a lack of open access information and methodological guidance targeted to public health professionals, particularly those based in low- and middle-income country (LMIC) settings. The objective of this review is to provide a comprehensive and accessible introduction to economic evaluations for public health professionals with a focus on LMIC settings. We cover the main principles underlining the most common types of full economic evaluations used in healthcare decision making in the context of priority setting (namely cost-effectiveness/cost-utility analyses, cost-benefit analyses), and outline their key features, strengths and weaknesses. It is envisioned that this will help those conducting such analyses, as well as stakeholders that need to interpret their output, gain a greater understanding of these methods and help them select/distinguish between the different approaches. In particular, we highlight the need for greater awareness of the methods used to place a monetary value on the health benefits of interventions, and the potential for such estimates to be misinterpreted. Specifically, the economic benefits reported are typically an approximation, summarising the health benefits experienced by a population monetarily in terms of individual preferences or potential productivity gains, rather than actual realisable or fiscal monetary benefits to payers or society.

## Introduction

Healthcare demand is continuing to grow; however, the resources available for healthcare are explicitly limited. Consequently, ensuring the best value for money spent in healthcare has been placed high on the agenda for governments worldwide, with economic considerations gaining an increasingly prominent role when planning, managing and evaluating health systems (1). Health economic analyses can be used to assess a health interventions value for money and can support the optimal allocation of the limited resources available for healthcare (2).

Using health economic analyses to investigate the value for money of different health interventions is particularly appealing to decision-makers when considering the use of a public budget to fund healthcare services. The role of health economic analyses in informing health policy has increased over time (3, 4). However, the underlining principles of the different types of health economic analyses are not always fully understood by non-health economists, and inappropriate types of analyses, as well as inconsistent methodologies, may be being used for informing health policy decisions, particularly in low- and middle-income country (LMIC) settings where this field of research is less well-established (5). This limits the potential role of these tools in informing policy decisions to lead to the greatest health gains. Although information and methodological guidance for these health economic analyses are available, it is predominantly in the form of textbooks and training programs, that are typically available in and focused on high-income country settings, and tend to be behind a paywall. Although many of the concepts are universally relevant, there are issues more specific to LMICs (such as the different types of data available, effectiveness metrics, and decision rules) and capacity in these settings is an ongoing challenge (5). Currently, there is a lack of up-to-date open access accessible literature focusing on a public health professional audience in LMICs who are playing key roles in health resource allocation.

This review provides a comprehensive, transparent, and freely accessible introduction to economic evaluations—intended to provide a resource for public health professionals, especially those in LMICs where such resources are typically unavailable. Specifically, we provide an up-to-date comprehensive introduction to the main principles underlining the most common economic evaluation methods used in the context of informing resource allocation decisions in global health [namely cost-effectiveness/cost-utility analyses and cost-benefit analyses (6)] (Table 1), outlining their key features, differences, advantages and limitations. By doing this we aim to increase the overall understanding of the key concepts underlining the different types of economic evaluations commonly used in the context of priority setting in healthcare, focusing on LMIC settings. Understanding the key concepts in this context is critical as it may influence the appropriate use of each approach as well as how health economic evidence should be interpreted to inform health policy. It is envisioned that this will help those conducting such analyses, as well as stakeholders that need to interpret their output [such as a cost per quality-adjusted life-year (QALY) gained, cost per disability-adjusted life-year (DALY) averted, net monetary benefit, return on investment, and benefit-cost ratio], gain a greater understanding of the approaches and help them choose/distinguish between the approaches. Note that this review focuses on the general application of these methodologies and is not focused on a particular disease area.

**Table 1:** The key types of full economic evaluations (6).

**Type**	**Description**
Cost-effectiveness analysis	Cost-effectiveness analysis is a form of comparative economic analysis that evaluates two or more policy alternatives in terms of their relative costs and outcomes, where the outcomes are measured in a single natural unit (e.g. life-years gained, disease case averted etc.).
Cost-utility analysis	Cost-utility analysis (a specific type of cost-effectiveness analysis) is a form of comparative economic analysis that evaluates two or more policy alternatives in terms of their relative costs and outcomes, where the outcomes are expressed by a generic measure of health status that considers both the effect on mortality and morbidity (e.g., quality-adjusted life-years (QALYs) and disability-adjusted life-years (DALYs)).
Cost-benefit analysis	Cost-benefit analysis is a form of comparative economic analysis that evaluates two or more policy alternatives in terms of their relative costs and outcomes, where both the costs and outcomes are expressed in monetary terms. In principle, it should value the interventions relevant costs and outcomes based on the preferences of those affected (i.e., the individuals' willingness to pay).
Cost-minimisation analysis	Cost-minimisation analysis is a form of comparative economic analysis that compares the costs of two or more policy alternatives which are all assumed to have equivalent health effects.
Cost-consequence analysis	Cost-consequence analysis is a form of comparative economic analysis that evaluates two or more policy alternatives in terms of their relative costs and outcomes, where the outcomes are not summarised in a single measure, and multiple outcomes of interest are reported.

## Differences in the Main Types of Health Economic Analyses Used for Evaluating Resource Allocation Decisions

There are many different approaches used for health economic analyses. Some types of analysis only examine the costs of an intervention or a disease (e.g., cost of illness studies) independently, whereas other types of analysis evaluate both the costs and consequences of an intervention. It is vital to understand the different roles of partial and full economic evaluations.

### Full Economic Evaluations vs. Partial Evaluations

**Figure 1:**
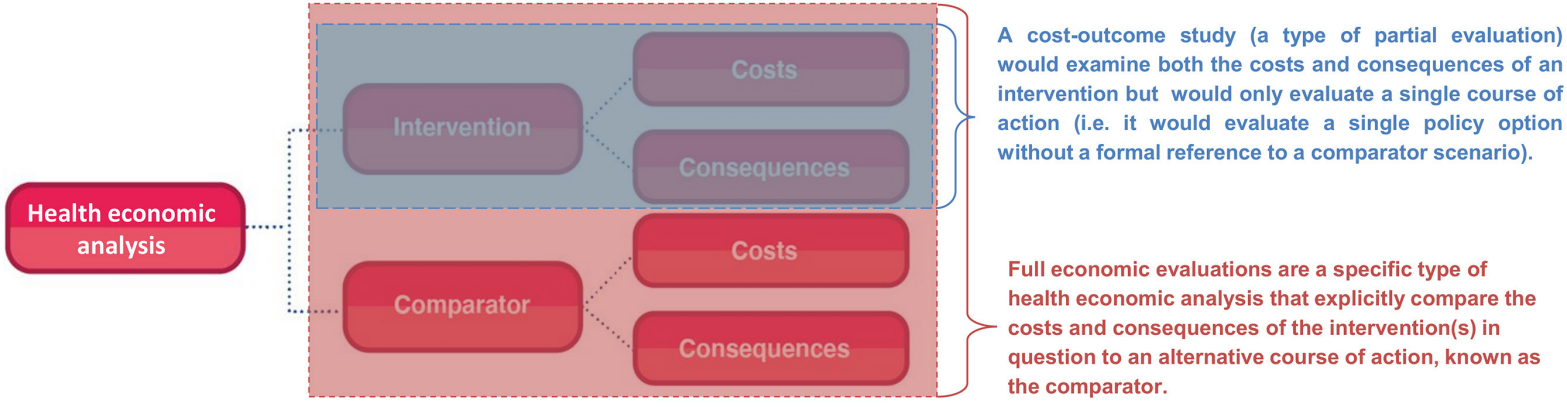
The difference between full economic evaluations and cost-outcome partial evaluations.

Full economic evaluations are a specific type of health economic analysis that explicitly compare the costs (use of resources) and consequences (effects) of the health intervention(s) in question to an alternative course of action, known as the comparator (Figure 1) (7). Full economic evaluations therefore formally evaluate at least two alternative courses of action, even when only looking at a single health intervention/policy. The comparator (also referred to as the counterfactual or baseline scenario) is typically chosen to reflect common practise or standard of care in the setting where the economic evaluation is undertaken. For example, an economic evaluation of introducing the human papillomavirus vaccine in the UK would use the pre-existing cervical cancer screening program as the comparator (8). In some cases, the comparator will be “do-nothing” or a no intervention scenario, but it is still a formal part of the analysis which needs to be clearly acknowledged with the relevant costs and consequences quantified.

In contrast, partial evaluations (also referred to as partial economic evaluations) are studies that either:

1. Only examine the costs or consequences of an intervention independently i.e. they do not look at both or relate the costs to the consequences. For example, a costing study would evaluate only the costs associated with an intervention (but not compare these to its consequences).
2. Examine both the costs and consequences of an intervention but only evaluate a single course of action (a cost-outcome study). By doing this they are either explicitly or implicitly ignoring the comparator, which may overlook relevant costs/consequences and does not allow for comparisons to relevant alternative policy options for that setting. These analyses are therefore not a full economic evaluation—which has to formally compare the costs and consequences of the interventions in question to a comparator scenario (Figure 1).

**Figure 2:**
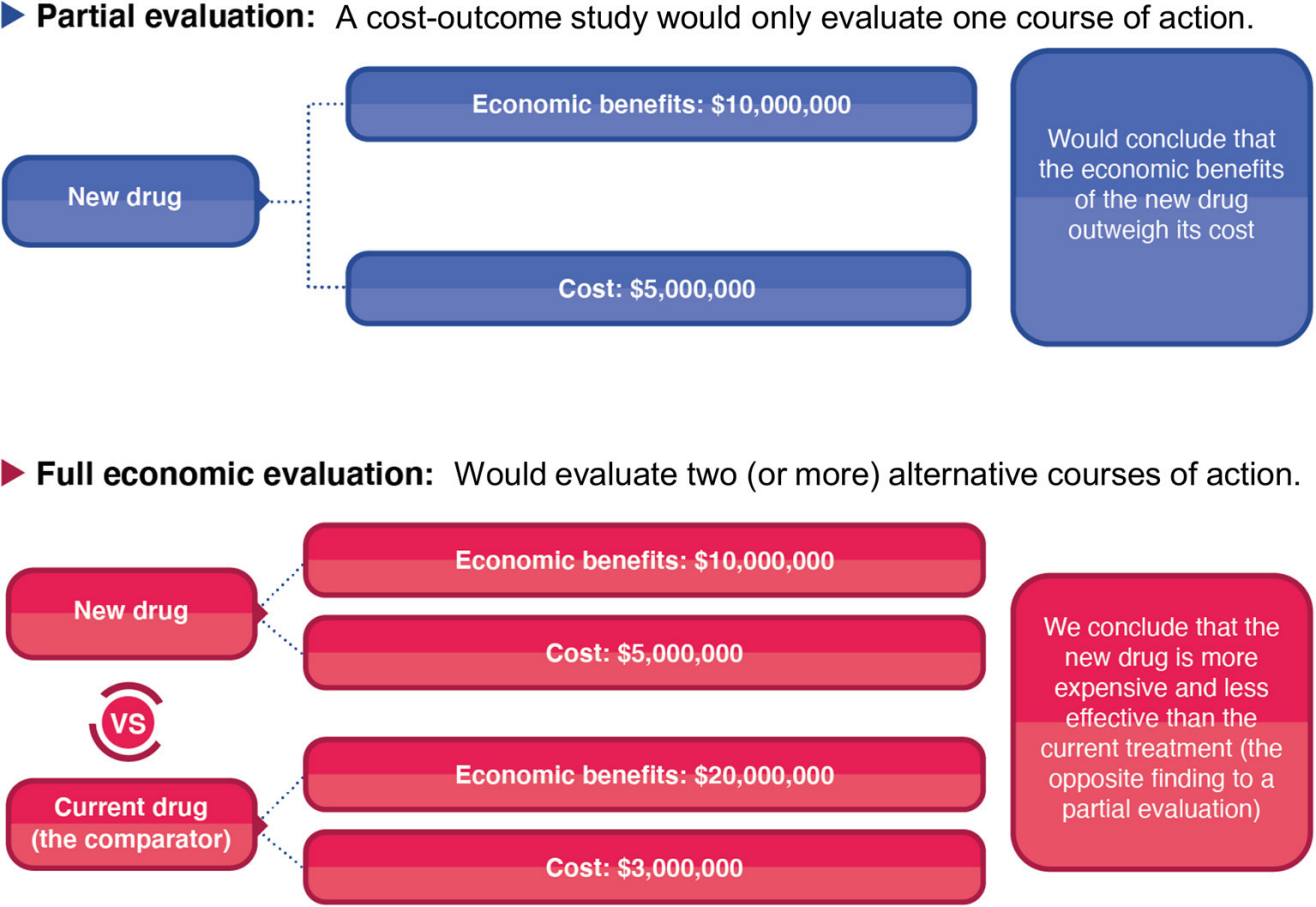
A hypothetical comparison of the difference between a partial evaluation and full economic evaluation.

Although partial evaluations can provide useful information, they cannot alone guide decision-making as only knowing an intervention's cost or the economic burden of a disease does not indicate an intervention's value of money. In the context of informing healthcare decision making surrounding resource allocation, it is vital to evaluate both the costs and consequences of the intervention in question and to compare it to a relevant alternative course of action/policy option (the comparator). The importance of accounting for the comparator is highlighted in Figure 2. In this hypothetical example, a partial evaluation would find that the benefits of a new treatment (the monetary value of its health benefits) outweigh its cost, favouring its use. However, performing a full economic evaluation whereby the new treatment was compared to the current standard practise as the comparator, the analysis would find that the new treatment is less effective and more expensive than the current standard practise—leading to the opposite policy recommendation (Figure 2). This highlights that when relevant alternative policy options (such as the standard of care) are ignored, analyses can generate misleading conclusions. Note that there is variation in the literature regarding if partial evaluations are referred to as an economic evaluation or not (9). Note that at times studies can be mislabelled. For example, Zarnke et al. (9) found that studies labelled as cost-benefit analyses (a type of full economic evaluation) in the healthcare literature were at times only partial evaluations (Figure 1).

### Different Types of Full Economic Evaluations

There are five main types of full economic evaluations used to inform and evaluate health interventions (outlined in Table 1) (6, 10). In this review, we focus on the most widely used types, i.e. cost-effectiveness/cost-utility analyses, and cost-benefit analyses (11, 12). There are other types of full economic evaluations, namely cost-minimisation analysis and cost-consequence analysis. As these are less commonly performed, and in the case of cost-minimisation analysis only appropriate in rare circumstances (11–13), these are not discussed in more detail within this review.

These different economic evaluations are based on similar principles. However, while they may first appear to be interchangeable, they differ in their fundamental methodology and interpretation. A key difference across the different types of full economic evaluation is how the outcome is expressed (Table 1). Cost-benefit analyses express the benefit or consequence of the health intervention in question in monetary terms (10, 14). In contrast, cost-effectiveness analyses measure the health consequences of the health intervention in a single natural unit (such as life-years gained, cases averted, or cases detected), and cost-utility analyses (a specific type of cost-effectiveness analysis) measure the health consequences using a generic measure of health status that considers the effects on both mortality and morbidity (10, 14), such as DALYs and QALYs (15). There is also variation in how these different analyses consider the efficiency of resource allocation (Table 2).

**Table 2:** A summary of how the different types of analyses consider the efficiency of resource allocation and examples of their use.

	**Cost-effectiveness analyses**	**Cost-utility analyses**	**Cost-benefit analyses**
How they consider the efficiency of resource allocation	Cost-effectiveness analyses using disease or programme specific metrics are often only concerned with how to use healthcare resources in a way that maximises their output for the cost. As such they are considering technical efficiency, identifying the option that provides the maximal health care for a given cost, or delivering a certain service at a minimal cost.^1^	Cost-utility analyses can consider the optimal allocation of healthcare resources (such as the mix of interventions) in a way that results in the maximum health gain for a given level of expenditure. In this way, it considers allocative efficiency within the health sector (i.e., it only deals with quasi-allocative assessments) (16).	Cost-benefit analyses can be used to consider the optimal allocation of resources in its broadest sense because once the benefits have been converted into monetary terms then the net economic benefit of different activities can be compared (including to those outside of the healthcare sector). It can therefore consider allocative efficiency across different sectors/across society.
Examples of when it is useful	Useful when a stakeholder is interested in a particular output metric and/or when you do not need to compare the outcome to other interventions/policy options.For example, comparing a range of different malaria interventions when investigating the cost per case averted.	Useful for informing health policy decisions when directly comparing different health interventions that fall within the same budget or benefit package (such as when considering the optimal allocation of a health care budget).For example, deciding if a new vaccine or treatment should be adopted within a national health benefit package.	Useful for cross-sectoral comparisons, such as looking at if other government spending should be reallocated to the healthcare sector (17, 18). Also useful when evaluating health policy/interventions where the health outcome metrics are not suitable (e.g., prenatal genetic screening for Down's syndrome (19)), and in certain complex intervention contexts (such as interlinked packages of care, where maximising health is not the only objective). For example, for considering non-pharmaceutical interventions for COVID-19 control in terms of both health and non-health outcomes.

*Note that cost-effectiveness analyses can be used to assess allocative efficiency within and potentially beyond the health sector for life-saving interventions when the outcomes are measured in terms of deaths averted or life-years gained, etc. (16)*.

Underlying the differences in how the outcome is expressed for economic evaluations are differences in the economic foundations. Cost-benefit analysis is traditionally based on a welfarist approach foundation, where the health outcomes are judged by the extent of their contribution to overall societal welfare based on the preferences of the individuals (their willingness to pay). In contrast, cost-effectiveness/cost-utility analysis are based on an extra-welfarist approach foundation, where the objective is traditionally to maximise contributions to societal health, measured as the sum of individual health status. An overview of these foundations is provided in Box 1.

Box 1 **Summary of the welfarist vs. extra-welfarist approaches to resource allocation.**

Under the welfarist approach, the efficiency of a health intervention is based solely on the individuals' perceived value of the welfare that results from it (known as individual utilities^*^). In functioning markets, social welfare based on individuals' preferences can be revealed through the market. However, due to market failures in health care, individuals' preferences must be elicited to value the consequences of implementing health care interventions (20, 21). These individual preferences are reflected by what the individuals are willing to pay (or give up) for the outcomes of the healthcare intervention in question; the higher their willingness to pay, the more the individuals prefer the consumption of these healthcare goods/services over alternative goods (22, 23). Under this approach, each individual is considered to be the best judge of their own welfare (23, 24) and social welfare is typically considered to only be a function of these individual preferences. Improvements in social welfare are judged in terms of a “potential Pareto improvement,” wherein a given change will be a potential Pareto improvement if individuals benefiting can compensate those made worse off whilst remaining better off (known as the Kaldor–Hicks criterion) (20, 25). However, this criterion is almost always applied hypothetically (i.e., no compensation actually needs to be paid) and hence some people do inevitably “win” while others “lose” (20). Here, health is often only taken into account insofar as it enables welfare to be derived from the consumption of healthcare goods/services (24) that is, only based on individual preferences. This approach is based on several assumptions regarding individuals' welfare maximising behaviour, including that individuals will make rational choices based on their preferences for the consumption of different goods, as well as that social welfare is only a function of such individual preferences (26).

The field of economic evaluation in health care has increasingly adopted an alternative framework, known as the extra-welfarist approach. The extra-welfarist approach was developed to adapt the classical welfare economic approach to the particular characteristics and context of priority setting within the healthcare sector (27). There are different interpretations of extra-welfarism, but in practise, they almost exclusively focus on the importance of health as the main outcome of health policies (27). Consequently, it has been described as introducing an important class of extra welfare sources that allows the consideration of other factors beyond individual utilities/preferences (22, 23, 28). Because under this approach the most important output of health services is considered to be health outcomes (28), information regarding different health status or health gains can be considered when evaluating interventions (24). Most extra-welfarists would argue that the “need for health care” and not individual demand, should be the priority for the allocation of resources within the health sector (27) and individuals' own judgements about their welfare are not necessarily the most important (24, 28). Extra-welfarism is based on the underlying idea that the assumptions regarding individuals' welfare maximising behaviour which underpin the welfarist approach, do not necessarily apply to health behaviours and healthcare (26, 29), and it rejects the exclusive focus on individual preferences when evaluating health interventions, allowing other factors to be considered (23).

*These concepts are discussed further in the following papers(**22*–*24**)*.

^*^Note that utility in this context is not the same metrics used with cost-utility analysis [such as quality-adjusted life-years (QALYs) and disability-adjusted life-years (DALYs)].

A limitation of this review is that it only discusses the most commonly used types of full economic evaluations. However, as well as full economic evaluations, there are other methods/frameworks used in priority setting in global health that include health economic analysis. For example, budget impact analysis examines the financial impact of the adoption and diffusion of an intervention within a particular setting, considering its affordability (30). Furthermore, multicriteria decision analysis (MCDA) is also used to assist policymakers in choosing between options where there are two or more relevant criteria (29) and can extend economic evaluation methods to consider other aspects such as acceptability, ethics and equity. Ochalek et al. (20) provide a summary of the methods/tools available for priority setting focusing on LMICs.

## Cost-Benefit Analyses

A cost-benefit analysis (also referred to as benefit-cost analysis) is a comparative analysis of the relative costs and outcomes of two or more alternative courses of action, where both the costs of an intervention and its resulting outcomes are expressed in monetary terms (31). In the past, it has been referred to by some as the gold standard economic evaluation approach (31, 32), and it is also used within other public sectors (17, 31). Cost-benefit analyses can be used to consider the optimal allocation of resources in its broadest sense because once the benefits have been converted into monetary terms then the net economic benefit of different activities can be compared (including to those outside of the healthcare sector). It can therefore consider allocative efficiency across different sectors/across society (Table 2).

Cost-benefit analyses are commonly summarised by estimating the net benefit of the intervention (the monetised benefits minus its costs), or by a benefit-cost ratio (the monetised benefits divided by its costs) (33). However, other outcome measures can also be used, such as the internal rate of return.

Conventionally, cost-benefit analysis involves summing the values of the costs and benefits of an intervention based on the preferences of those affected (34). The theoretical foundation of cost-benefit analyses for healthcare is based on the welfarist approach (Box 1). Conceptually, this is based on three elements (18). The first is that each individual is the best judge of their own welfare (or well-being) (18). The second is that measures of welfare are what is important when measuring health, and these are based on the utility (or perceived value) individuals' receive from the consumption of healthcare goods/services (29). The third is that the preferred policy/intervention is that which maximises social welfare, that is, the reallocation of resources for an intervention are justified as long as the net benefits increase (or the gains can hypothetically fully compensate costs or losses) (18). The framework does not typically take into account “who” is benefiting or losing, and no compensation need actually be paid (Box 1). For functioning markets, social welfare based on individuals' preferences can be revealed through the market (21). However, due to market failures in health care, individuals' preferences must often be elicited using other methods (20). In practise, various methods are used to quantify the health consequences in monetary terms, including willingness to pay (Box 2) and valuing productivity gains (Box 3). It is debatable how many analyses fit within these original underpinnings when being applied to healthcare decision making.

Box 2 **Willingness to pay.**

The willingness to pay technique is based on the premise that the maximum amount an individual is willing to pay (or sacrifice) for a given commodity is an indicator of its “value” to them (35). Using the willingness to pay technique in this context could involve estimating what an individual is willing to pay for certain health benefits, consequently estimating the value of the health benefits of an intervention in monetary terms for that individual. An advantage of this is that it is argued that when an individual is considering their maximum willingness to pay, they will take account of all the attributes of the service of importance to them, not just the health gains (35). Willingness to pay estimates are usually derived using either stated or revealed preference methods. Stated preference methods typically use survey questions to ask respondents about their willingness to pay for an outcome within a hypothetical scenario (33). In contrast, revealed preference methods indirectly infer the value of nonmarket outcomes (such as health benefits) from observed behaviours or prices for related market goods (33). For example, some individuals accept extra income for work that is associated with an increased risk of injury or death or spend more money on items that have enhanced safety features (31). When the extra income received or increased expenditure is compared to the change in the degree of risk associated with a particular activity, it is possible to establish the individuals' personal valuations implicit in the observed behaviour (31). An example of a willingness to pay metric is the “value per statistical life” which captures how much individuals are willing to pay to reduce the risk of death and is used to estimate a monetary value on reductions in mortality. This is often based on trade-offs individuals are willing to make between fatality risk and wealth. It is important to note that the term is often misunderstood or misinterpreted as the value the government or the researcher places on saving a life (36), which is a fundamentally different interpretation/a different type of output.

Willingness to pay methods are associated with several limitations in the context of healthcare:

- Willingness to pay estimates are sensitive to the survey methodology and how the questions are phrased (37, 38). In addition, an individual's willingness to pay is inevitably correlated to their ability to pay and therefore willingness to pay estimates may be affected by the individuals' wealth, income and social status (31, 39–41). Consequently, it has been argued that monetary valuations of health consequences based on willingness to pay methods leads to evaluations intrinsically favouring interventions against diseases of the wealthy over those of the poor (40, 41). In addition, both stated preference and revealed preference methods have their own specific limitations:
- Stated preference methods in this context are based on a number of assumptions, including that individuals' will make rational choices when valuing different health outcomes (42, 43). However, the choices given within questionnaires are hypothetical and there is little need for a respondent to think carefully about what they would choose in a real situation, or to report accurately (33). In addition, when individuals respond to these questionnaires (especially those asking about unfamiliar outcomes, involving small probabilities and long-time horizons), they tend to ‘construct' their preferences on the spot in response to context-specific stimuli (42, 44). Responses are therefore vulnerable to psychological biases (42, 44). Furthermore, unlike when buying private goods, where consumers gather information, compare it to similar goods and actively evaluate their preferences, they may not have all of the information and knowledge necessary to rationally value different health outcomes.
- Revealed preference methods indirectly estimate individuals' willingness to pay by inferring the value of a health outcome from observed behaviours or prices for related market goods. However, individuals may not have perfect knowledge of the risks associated with the observed behaviours in question, and the estimates may not be representative of the risk preferences of the overall population (45), potentially biassing the willingness to pay estimates. Revealed preference methods are often used to estimate the value per statistical life metric. However, far fewer studies are conducted in low- and middle-income countries (LMICs), and the quality varies widely (17). Consequently, value per statistical life estimates often to need to be extrapolated to LMICs settings (39), potentially generating substantial uncertainty in the resulting output.

Box 3 **Valuing productivity gains.**

The valuing productivity gains (or human capital) approach involves placing a monetary value on the estimated productivity losses associated with a disease that are averted due to a health intervention. However, this is associated with several challenges (note that these also apply when including productivity gains within cost-effectiveness/cost-utility analyses):

- Quantifying productivity losses or gains accurately is difficult and identifying the appropriate unit to measure is challenging. The approaches used for this can give very different outcomes (Figure 5). For example, within the human capital approach, all potential production not performed by a person because of morbidity or early mortality is counted as a production loss (41, 46, 47). In contrast, within the friction cost approach, the calculated production losses are limited to the time needed to replace an ill employee and train a new employee (friction period) i.e. only the productivity lost by an ill employee before they are replaced (41, 46, 47).
- In addition to how the productivity losses are quantified, how they are valued is also variable (48). For example, whether/how to include taxes, employer-paid benefits, how to capture labour market constraints on how much work an individual does and whether to adjust for future increases in wages are issues subject to debate (48). This variation in how the productivity costs are quantified and valued leads to inconsistencies between studies (49).
- Valuing productivity gains can discriminate against those who receive lower wages and those not in the workforce (such as children and the elderly). This is made worse by the fact that the productivity losses/gains related to unpaid work (such as housework and voluntary work) are difficult to quantify and are often ignored (50).
- This approach has been criticised for not being consistent with the theoretical foundations of cost-benefit analyses in welfare economics, as it focuses on changes in productivity rather than measuring overall social welfare and is not based on an individual's personal valuation of the benefits (31, 51, 52).

Within cost-benefit analyses, the decision rule for selecting an optimal intervention is if its net benefit is positive or its benefit-cost ratio is above one i.e. if its monetised benefits outweigh its costs, then the intervention is deemed justified in terms of increasing societal welfare. Importantly, money or cost savings are not the primary focus (33). Instead, it typically indicates the trade-offs individuals are willing to make between spending on the health intervention outcomes (such as improved health) compared to other goods and services (33)—at least when based on willingness to pay estimates. Although formally a cost-benefit analysis should be based on a welfare economic foundation, the term is often simplified to studies with both costs and outcomes expressed in a monetary form.

There are other health economic analyses that express the benefit of a health intervention in monetary terms, such as return of investment analysis and social return of investment analysis. When these formally evaluate the costs and consequences of an intervention compared to a comparator or counterfactual scenario (i.e., look at two or more alternative courses of action), they are a full economic evaluation and are similar to a cost-benefit analysis and have many of the same advantages/limitations (Boxes 2, 3). However, when these only formally evaluate the costs and consequences of a single policy option and do not appropriately account for the comparator or counterfactual scenario, they are only a partial evaluation (a cost-outcome study) (Figure 1). Although these are often referred to as an extension of cost-benefit analysis, these analyses are less well defined.

### Cost-Benefit Analyses: Advantages

#### More Consistent With How Other Public Interventions Are Evaluated and Facilitate Cross-Sectoral Comparisons

The framework used within cost-benefit analyses is more consistent with how other public interventions/policies (not only within the health sector) are evaluated, i.e., based on how much individuals are willing to trade for the benefits (17, 31). Cost-benefit analyses can therefore be used to consider the optimal allocation of resources in its broadest sense because once the benefits have been converted into monetary terms then the net economic benefit of very different activities can be compared, including to those outside of the healthcare sector (53). For example, the “gain” to society from a new health policy can be compared to investing in transportation infrastructure or education (53). This facilitates cross-sectoral comparisons and allows consideration of the allocative efficiency of resources across different sectors/society (Table 2). In contrast, cost-utility analyses typically only consider the allocative efficiency of resources within the healthcare sector (Table 2).

#### Potential to Value a Wider Range of Benefits

This type of analysis can incorporate benefits and costs that fall outside of the healthcare sector. This can capture potential benefits of interventions missed by cost-effectiveness/cost-utility analyses.

#### Monetary Output Is Desirable to a Range of Stakeholders

A monetary output tends to be desirable to both healthcare and non-healthcare decision-makers (such as those of the ministry of finance and patient representatives), as well as the public. Also, by placing 'proxy' monetary values on all identified impacts of interest to the stakeholders they can account for multiple stakeholders' views of impact simultaneously. This facilitates the stakeholders' voice in resource allocation decisions.

### Cost-Benefit Analyses: Limitations and Areas of Debate

#### Methodological Difficulties Regarding Placing a Monetary Value on Health Benefits and Variations Between Studies

There is no consistently agreed-upon gold standard method for placing a monetary value on the health benefits of interventions. Consequently, although they have the same monetary outcome, the approaches used can be highly variable—making it difficult to compare different studies (51). The different main approaches and their associated limitations are outlined further in Boxes 2, 3.

A comparative analysis of the cost-benefit analyses of vaccination found that applying different approaches to monetise health benefits in cost-benefit analyses can lead to widely varying outcomes (51). The variation in the approaches taken (and their quality) is important as there is always going to be a need to compare different health economic analyses informing resource allocation. In addition, the variation in approaches could lead to biases in the results of health economic analyses and subsequently, the health policies/decision making they inform. We would argue that the greater the variation between the different studies the less useful they are for practically informing resource allocation within global health. Recently, a reference case for cost-benefit analysis has been developed (33). These guidelines will hopefully bring much-needed consistency in this area.

#### Difficulties Capturing Non-fatal Health Outcomes

Willingness to pay estimates are less common for non-fatal health outcomes, particularly within LMICs. Hence, many studies in this area use averted cost or averted cost-of-illness estimates as a proxy to quantify the monetary benefits of non-fatal health outcomes of health interventions (33) (i.e., they look at the averted direct costs and productivity costs that are associated with a condition or treatment). However, the quality of such cost/cost of illness studies is variable (54, 55). This makes it harder to apply a cost-benefit analysis for evaluating interventions whose main health benefits are not related to averted excess mortality. In addition, although this is a pragmatic solution to limited data, it could be argued that it does not fit within cost-benefit analyses welfarist approach underpinning.

Another method is to use a willingness to pay threshold to convert DALYs averted or QALYs gained into a monetary value (33). Although this is increasingly being used, it has been argued that these conversions have no theoretical basis and can be very arbitrary (39).

#### Potential Misinterpretation and Difficulties Regarding the Practical Interpretation

The results of these studies can have the potential to be misinterpreted. Specifically, these types of studies are typically estimating measures of social welfare or potential economic benefits (not estimating realisable financial benefits). This means that the economic benefits being reported are typically an approximation as they are a way of summarising the health benefits experienced by a population in monetary terms (which can be based on the approximated monetary value of estimated productivity gains or willingness to pay methods). For example, the human capital approach would be valuing potential productivity gains and not the productivity gains actually experienced by the population and the willingness to pay technique would be valuing the benefits based on the individuals' preferences and how much they would be willing to pay to obtain the health gains. The risk here is that many stakeholders may not realise how intangible these estimated economic benefits can be and may misinterpret them as the actual monetary benefit to society (i.e., real or realisable monetary benefits). For example, if the estimated benefit-cost ratio of an intervention is 6.0, this can be misinterpreted as the intervention generating six times the amount spent on it (in the same way as a financial investment portfolio might be interpreted). However, not all the estimated economic benefits are realisable, so it may not generate cost savings at all. This is not to say that cost-benefit analysis is not meaningful but greater care is needed in how these studies are reported and interpreted. Importantly, the goal of such analyses is typically to determine if an intervention is justified in terms of increasing societal welfare and if the estimated economic benefits outweigh the cost of the intervention, not if it generates fiscal cost-savings.

Similarly, when a study reports that the economic benefits outweigh the costs of an intervention it can be referred to as having a positive return on investment. However, this may be misleading to policymakers as the reported return on investment of public health interventions is increasingly being misunderstood as a synonym for interventions generating “cost-savings” (39). For example, a systematic review reported that the median return on investment for public health interventions was 14.3 to 1 (56). Many misinterpreted this to mean that for every £1 spent, public health interventions will save the public sector £14.30 in cash; implying a cash return of 1,430% (39). However, this is not the correct interpretation as not all of the economic benefits calculated will be fiscal cost-savings (39).

A further limitation is that there is no threshold (marker or cut off point for when the perceived costs outweigh the perceived benefits) for which an intervention is considered to be of good value in a particular setting. The decision rule of whether to implement the intervention under consideration is based on whether the benefits outweigh the costs (i.e., if the benefit-cost ratio is above one) (20). Typically, cost-benefit analyses do not account for issues surrounding fixed budget constraints for particular sectors (e.g., health) and therefore fail to accurately reflect the opportunity costs of additional costs being imposed on these budgets and what other health interventions may need to be displaced (20).

#### Equity and Distributional Concerns

Cost-benefit analyses involve placing an economic value on averted mortality and morbidity. However, this can have implicit equity and distributional concerns and many have argued that such evaluations will intrinsically favour health interventions benefiting richer over poorer populations (40, 41, 57). For example, when aiming to quantify the economic benefits experienced by a group of patients, all things being equal, the value of the increased social welfare or productivity gains of averting disease in richer populations will be higher than poorer populations within the same country/setting (58, 59). In addition, the economic benefits of treating women for a condition (who have a higher burden of unpaid work and on average lower salaries) could easily be estimated to be lower than for treating men, implicitly implying that interventions targeting diseases in men have a higher value than for women (58, 59). A way around this issue is to assume the same value of time or life for everyone (regardless of gender, social-economic status, and employment). However, it could be argued that doing this means the estimated economic benefits are more hypothetical and have a less obvious meaning i.e. if you use the same value of time for everyone, what do the estimated economic benefits actually correspond to. Again, the problem is that it is not necessarily always being understood that the economic benefits being presented are often an approximation/theoretical and not necessarily directly realisable to the setting's society/economy.

It should be noted that attempts have been made to adapt cost-benefit analyses to address these equity and distributional issues (33, 34, 60). However, it is yet to been seen if these will become operationalised into policy-oriented cost-benefit analyses within global health.

## Cost-Effectiveness and Cost-Utility Analyses

Cost-effectiveness/cost-utility analyses are some of the main types of economic evaluations used for healthcare (11). They are a comparative analysis of the relative costs and outcomes of two or more alternative courses of action. Cost-effectiveness analyses measure the health consequences of an intervention in a single natural unit (such as cases averted, or life-years saved). However, a limitation of this is that it is difficult to compare studies investigating interventions targeting different diseases or different stages of care, since their health consequences will be expressed in different units, limiting its potential use for informing policymakers. To address, this a specific form of cost-effectiveness known as cost-utility analysis was developed. Cost-utility analyses measure the health consequences with a generic measure of health status, which can account for benefits on both reduced morbidity and mortality, such as DALYs and QALYs (61). As these metrics can be used for a wide range of diseases, the cost-effectiveness estimates for different healthcare interventions can be directly compared to each other (such as comparing the cost-effectiveness of a malaria intervention to a tuberculosis intervention). In practise, there has been a blurring of the distinctions between cost-effectiveness and cost-utility analyses; as a result, literature on cost-effectiveness often encompasses both these approaches and cost-utility analyses are often referred to as a cost-effectiveness analysis (16). Cost-effectiveness/cost-utility analyses are typically based on the extra-welfarist approach (Box 1), where the objective is traditionally to maximise contributions to societal health, measured as the sum of individual health status.

**Figure 3:**
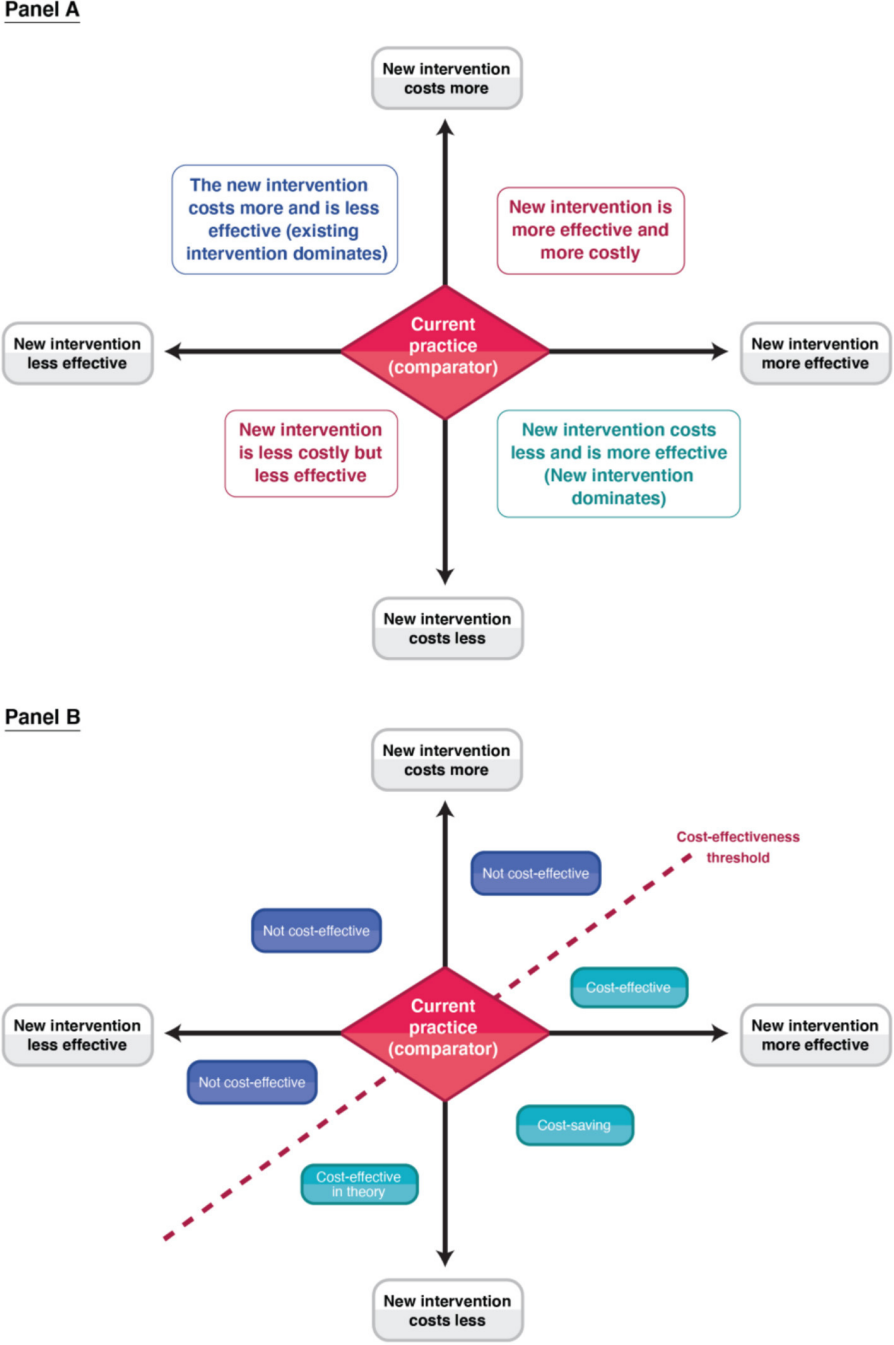
A schematic of the cost-effectiveness plane. Panel A indicates the four different quadrants. Panel B illustrates the different decision rules in relation to a cost-effectiveness threshold within the different quadrants.

Figure 3 provides a visual representation of what can happen when comparing a new health intervention to a comparator within a cost-effectiveness/cost-utility analysis [outlined further in (62)]. When the analysis is using a generic measure of health status (such as DALYs/QALYs within cost-utility analyses), standardised cost-effectiveness thresholds (an explicit cost per unit of outcome below which an intervention is considered cost-effective—such as £20,000 per QALY gained) are frequently used as a decision rule to class whether an intervention is cost-effective or not (Figure 3). Such thresholds can be determined using a variety of methods (63). When this is done, the analysis can consider the allocative efficiency of resources within the healthcare sector (Table 2) (i.e., the allocation of healthcare resources in a way that results in the maximum health gain for a given level of expenditure) (16, 53). As such, when looking at mutually exclusive policy options (such as different treatment options for tuberculosis), the analysis considers the options incrementally and identifies the most effective option that is below the settings cost-effectiveness threshold (which is not necessarily the same as the option that has the lowest cost per unit of effect). In contrast, when a cost-effectiveness analysis uses a disease or programme specific outcome measure (such as cases averted), it is rare to have a standardised cost-effectiveness threshold available to compare the results to and it is difficult to compare the results to other studies. In this case, the goal of the analysis is often to identify the most efficient option in terms of the lowest cost per unit of effect i.e., identify the option with the lowest cost-effectiveness ratio (such as the lowest cost per case averted). In this context, cost-effectiveness analyses using disease or programme specific metrics are often only concerned with how to use healthcare resources in a way that maximises their output for the cost (known as technical efficiency (Table 2) (16, 53).

The outputs of cost-effectiveness/cost-utility analyses are typically expressed in ratios (a cost per unit of effect). It is also possible to express the outputs of cost-utility analyses in monetary terms (such as net monetary benefit) (20, 64). With net monetary benefit, the health outcomes are monetised based on a cost-effectiveness threshold which represents a societal valuation of a health metric, rather than an individuals' willingness to pay for health gains. Hence, although this is similar to a cost-benefit analysis, it does not have the welfarist foundation and uses a different discussion rule (Box 1).

Note that there are other specific forms of cost-effectiveness analysis used for priority setting, such as distributional cost-effectiveness analysis and extended cost-effectiveness analysis. These are described in more detail in (20, 65–67).

### Cost-Effectiveness and Cost-Utility Analyses: Advantages

#### Focus on Societal Valuation of Health

The framework behind cost-effectiveness/cost-utility analyses is the most appropriate if you accept that health is a fundamental good whose value should be determined by society, regardless of how much individuals are willing to exchange for it (i.e., regardless of their willingness to pay). This is more consistent with the theory of health rights that World Health Organisation (WHO) advocates for (68). In addition, it avoids the difficulties in valuing social welfare that occur due to market failure in the health care market.

#### More Commonly Performed

Cost-effectiveness/cost-utility analyses are a more commonly conducted and published method compared to cost-benefit analyses (11). This makes it easier for policymakers to compare different studies and interventions, facilitating the decision-making process. However, the comparability of cost-effectiveness analyses using disease or intervention specific metrics is difficult between different health programmes/interventions and it cannot be used to compare programmes with different aims/outcomes; which is why cost-utility analysis was developed.

#### Forgoing the Need to Monetize Health Benefits

The framework does not require assigning a monetary value on health gains. By foregoing this step, the analysis draws attention exclusively to health benefits and avoids the corresponding ethical and equity issues that can arise when monetizing them (69). However, it could be argued that some of these ethical and equity issues still arise when setting the cost-effectiveness threshold.

#### Guidelines for Reporting and Methodology

There are more established guidelines/reference cases for conducting and reporting the results of cost-effectiveness/cost-utility analyses within the health sector and in some settings, country-specific guidelines have been established (70–73). Although we would argue that the methodology used is more standardised compared to that of cost-benefit analyses (51), it should be noted that methodological variation still occurs between different cost-effectiveness/cost-utility analyses (such as the costs that are included, the perspective, and the assumed discount rate) (74, 75), particularly for settings that do not have standardised guidelines/reference cases.

### Cost-Effectiveness and Cost-Utility Analyses: Limitations and Areas of Debate

#### Availability of Cost-Effectiveness Thresholds

A cost-effectiveness threshold is often used as a decision rule to class whether an intervention is cost-effective or not. Some countries have established their own cost-effectiveness thresholds (89, 90) but this is rarer for low and lower-middle-income countries. The absence of an established threshold can make the results of cost-effectiveness/cost-utility analyses more difficult to interpret. In the past, when no country-specific threshold has been set many used the cost-effectiveness thresholds set by the Commission on Macroeconomics and Health (91); namely a cost per DALY averted <3 and <1 times the country's gross domestic product (GDP) per capita for a intervention being cost-effective and highly cost-effective, respectively. However, these are now considered to be too high and have become widely criticised (63, 92–95). The WHO has outlined that these thresholds were not intended for individual investment decisions but as a broad principle for global/regional level analyses (96). They have argued that these thresholds have therefore not been used in the way they were originally intended (96). However, unfortunately, these 1-3 times GDP per capita thresholds are currently still being used within global health (95). Although guidance exists, determining a country's cost-effectiveness threshold remains a complex area (93, 97, 98).

Depending on how it is calculated, the cost-effectiveness threshold can be a way of capturing how much health is expected to be foregone if the resources in question are used for an intervention (the health opportunity cost). When this is the case, the cost-effectiveness/cost-utility analysis can account for the budget constraints within the healthcare sector (99). However, in practise, the cost-effectiveness thresholds commonly used in LMICs do not account for this and are often only an expression of value by a specific party (such as an international organisation or government), without consideration of health care system constraints (99) (such as the 1 and 3 times GDP per capita thresholds previously mentioned). Revill et al. (99) discuss these concepts further in the context of priority setting in global health.

**Table 3:** Summary.

	**Cost-benefit analysis**	**Cost-effectiveness/cost-utility analysis**
Summary	A comparative analysis of two or more alternatives in terms of their relative costs and consequences. Within a cost-benefit analysis, both the costs and outcomes are expressed in monetary terms. This type of analysis typically involves summing the values of an intervention's costs and benefits based on the preferences of those affected. The analysis foundation is based on the welfarist approach (Box 1).	A comparative analysis of two or more alternatives in terms of their relative costs and consequences. Within a cost-effectiveness analysis, the health consequences of a health intervention are measured in a single natural unit. In contrast, a cost-utility analysis is a specific form of cost-effectiveness analysis, where the health consequences are measured using a generic measure of health status that considers both the effect on mortality and morbidity (such as DALYs/QALYs). The analysis foundation is based on the extra-welfarist approach (Box 1).
Advantages	More consistent with how other public interventions are evaluated and the facilitation of cross-sector comparisons (i.e., can compare the economic benefit to investments outside of the healthcare sector). It can therefore consider allocative efficiency of resource allocation across different sectors/society (Table 2). Potential to value a wider range of benefits. A monetary output is desirable to a range of stakeholders.	More commonly performed-making comparisons of different studies easier.More established guidelines for methodology and reporting.Forgoes the need to monetize health benefits and the corresponding equity and ethical issues.The cost-effectiveness threshold^a^ can be a way of capturing how much health is expected to be foregone if the resources in question are used for an intervention accounting for the budget constraints within the healthcare sector.
Limitations and areas of debate	Methodological and data difficulties regarding placing a monetary value on health benefits – particularly to non-fatal health outcomes. Methods used are more variable and there are less well-established reporting guidelines – making it harder to compare studies. Output can easily be misinterpreted and difficulties regarding practical interpretation. Less commonly performed than cost-effectiveness/cost-utility analysis. Placing a monetary value on health benefits generates notable equity concerns. Typically, do not account for issues surrounding fixed budget constraints and therefore fail to accurately reflect what other health interventions may need to be displaced.	Limited availability of cost-effectiveness thresholds.DALY and QALY generic summary measures have limitations.Non-health benefits may not be fully captured and there is variation regarding if/how they are quantified and included.Difficult to compare the results to investments outside of the healthcare sector (limited potential for cross-sector comparisons). They have a more limited potential in how they can consider the allocative efficiency of resource allocation particularly beyond the healthcare sector (Table 2).Although cost-effectiveness/cost-utility analyses avoid the need to monetize health benefits, they are still associated with equity and ethical issues.
Key message for policymakers	Cost-benefit analyses facilitate cross-sectoral comparisons and can include a wider range of benefits. However, there needs to be greater awareness of the current methods and variations in the approaches being used when placing a monetary value on health benefits (Boxes 2, 3), and the potential for the results to be misinterpreted. Importantly, the economic benefits being reported within such studies are typically an approximation, in terms of social welfare or potential economic benefits related to productivity gains. The risk here is that many may not realise how intangible these estimated economic benefits can be and may misinterpret them as the actual realisable or fiscal monetary benefits.	Cost-effectiveness/cost-utility analyses have key advantages for directly comparing different health interventions when considering resource allocation within the healthcare sector. However, it is important to acknowledge that notable non-health benefits of interventions may not be captured. Although some of these broader benefits can, in principle, be monetised and included in analyses when using a broad societal perspective, there is variation in when and how they are included. It is also more difficult to compare the results of cost-effectiveness/cost-utility analyses to investment in other sectors, limiting cross-sector comparisons.

a *Depending on how it is calculated*.

#### The Generic Measures of Health Status (DALYs and QALYs) Have Limitations

The use of DALYs or QALYs as outcomes within cost-utility analyses has been a notable improvement compared to only measuring the intervention's effect in terms of disease cases or deaths averted, and these measures permit comparison across different diseases or interventions. However, these generic measures of health status have limitations (Box 4). A general limitation of these generic summary measures is that they could be argued to be overly simplistic and reductionist and may not be capturing all of the health benefits of an intervention (80, 84). In addition, these metrics do not work well in certain situations, such as when health outcome metrics are not the most suitable. For example, DALY/QALY metrics could be argued to fail to account for the complexities of investigating the benefits of prenatal genetic screening and pregnancy termination for Down's syndrome (such as the impact on caregivers) (19).

Box 4 **Limitations of DALYs and QALYs.**

DALYs: The universal disability weights used within DALY calculations do not account for how the local context may influence the burden of a disease (such as the impact of living in poverty) (76, 77). Within the updated global burden of disease study (GBD) framework (post-GBD 2010), the disability weights are intended to solely be measures of losses of “optimal health” and are not intended to represent losses of well-being/welfare (78, 79). Consequently, the DALY disability weights do not consistently/fully account for the psycho-social implications of a disease or its sequelae (80) and its overall impact on quality of life. Critics have argued that disease burden should be quantified in terms of overall welfare loss and that only measuring burden as “lost health” may lead to biases when estimating the disability weights (78, 81, 82). Additionally, although the disability weights are meant to be standardised for a given disease, there is still variation in what weights are used in practise (27, 83). Furthermore, in comparison to preference-based health-related quality of life measures, DALYs may not fully capture the relative benefits of interventions that reduce the functional burden of a condition without curing it.

QALYs: QALY weights are typically estimated for a particular setting. A variety of tools/questionnaires can be used for this (84) (such as the EQ-5D questionnaire); however, it has been shown that the different utility measurement tools provide variable results (85) and the outcomes are sensitive to the survey design and sample (86). This can lead to variation in the utility weights being used by different studies. The generic instruments used to generate QALY utility weights are also insensitive to some medical conditions (84) and have also been critiqued for having insufficient sensitivity to measure small but clinically meaningful changes in health status (86). There is also ongoing debate regarding who should value the health states (patients with the condition or the general population) (84, 87), and this variation in methodology can lead to inconsistencies in how QALY utility weights are estimated. In addition, the financial barriers for accessing QALY weight estimation tools and databases disproportionately affect low- and middle-income countries (LMICs) (88).

**Figure 5:**
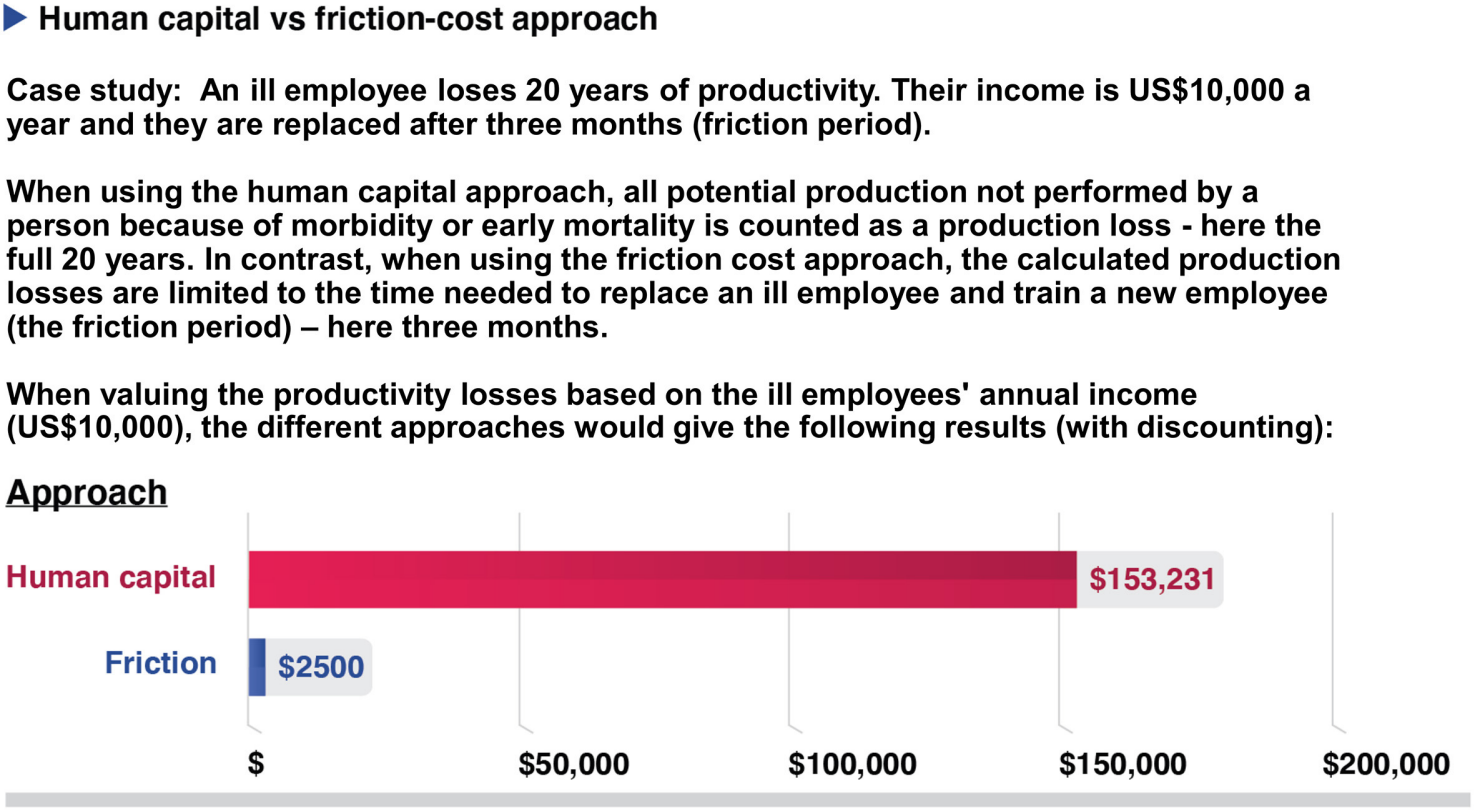
A hypothetical example showing the potential difference between the human capital approach or the friction cost approach when estimating productivity costs. Costs are being discounted at 3% per year.

#### Often Do Not Capture Non-health Benefits and Limited Cross-Sector Comparisons

The effectiveness framework of cost-effectiveness/cost-utility analyses focuses on measuring the benefits of public health interventions in terms of health gains (69, 100). When an intervention leads to cost-offsets/health savings (such as averted future medical costs), these cost-savings can be included and are subtracted from the cost of the intervention. However, many health interventions generate important additional benefits to other sectors, such as the environment or education (69, 100). Although all financial and some non-financial broader benefits can, in principle, be monetised and included in cost-effectiveness/cost-utility analyses when using a broad societal perspective, there is variation in when and how they are included (101). Consequently, many cost-effectiveness/cost-utility analyses only consider the benefits to the health sector and the impacts of public health interventions on other sectors are often not included (100). In some cases, this restrictive perspective is linked to the mandate for the healthcare budget, such as for solely improving health. Not capturing these non-health benefits could be undervaluing the broader benefits of many health interventions (39).

This limitation is particularly relevant for the evaluation of complex interventions (such as those involving interlinked packages of care) as the complexity means the intervention may not fit into one of the current appraisal systems, and maximising health may not be the intervention's only objective (102).

Notably, the decision in question may not only involve the health sector and can involve trade-offs between health and other sectors/societal aims. Using non-monetary metrics limits potential cross-sector comparisons, that is, it is hard to directly compare the estimated value of investing in a health intervention (based on the cost per DALY averted or QALY gained) to investing outside of the health sector, such as in education. This makes it difficult to use this type of analysis to justify the reallocation of other government spending to the health sector (such as adjusting tax policies) and more difficult for decision-makers to compare the value of money for a broad range of potential activities, including those from non-health sectors.

Generally, cost-effectiveness/cost-utility analyses have a more limited potential in how they can consider the efficiency and resource allocation compared to cost-benefit analyses (Table 2).

There is increasing recognition that the maximisation of health gains is not the only factor that individuals are concerned about in relation to healthcare decision making, as DALYs/QALYs may not be capturing the full consequences of interest when evaluating different interventions (24). Consequently, the importance of reporting other metrics/outcomes as well as DALYs/QALYs is becoming recognised (24, 29).

#### Variation in the Inclusion of Productivity Costs and Potential Double-Counting of Effects

**Figure 4:**
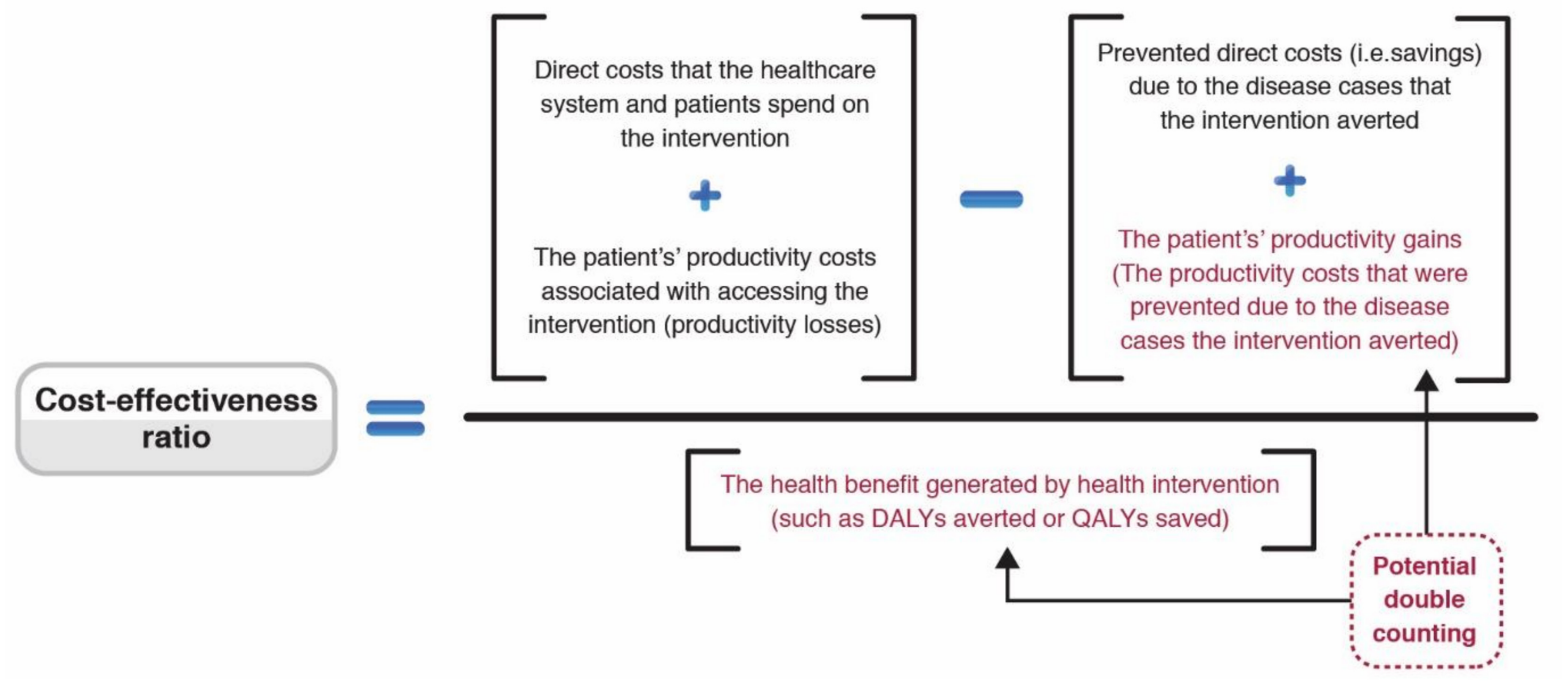
Schematic surrounding the inclusion of productivity costs within cost-effectiveness ratios. Adapted from (49).

There is variation regarding what types of productivity costs are included within cost-effectiveness ratio calculations (Figure 4). While including the productivity costs that are related to the patients' productivity losses associated with accessing an intervention is relatively uncontroversial when using a societal perspective, many have argued that those related to the productivity gains associated with prevented morbidity/mortality should not be included (10, 46, 103–107) (Figure 4). This is because these benefits are arguably being captured within the QALY/DALY effectiveness measure. Therefore, including these productivity gains within the cost part of the equation would potentially lead to the double-counting of the effectiveness of the health intervention (Figure 4) (41). However, this recommendation has been challenged with some arguing that the QALY measure does not capture these productivity gains (10, 103–107). Guidelines regarding the inclusion of these productivity gains vary (72, 74).

As previously highlighted, estimates of productivity costs are highly sensitive to the method used (48) and it is important to be aware of the potential variation in methodology when comparing studies (Box 3). This variation could lead to potential biases in setting health policy and further guidance is needed.

#### Equity

It should be noted that although cost-effectiveness/cost-utility analyses avoid the need to monetize health benefits, they are still associated with equity and ethical issues (40, 58, 100, 108) (such as those associated with the DALY/QALY metrics and the setting of the cost-effectiveness threshold). For example, cost-effectiveness/cost-utility analyses rarely provide information about who gains and who loses from health interventions (i.e., are equity neutral) and therefore do not prioritise improving health among the poorest even though this may be a societal objective. There have been new efforts to incorporate equity in cost-effectiveness/cost-utility analyses as a quantitative component which may potentially help with partly addressing these limitations, such as the development of distributional cost-effectiveness analysis and extended cost-effectiveness analysis (65–67). However, this is still an area that needs attention regarding practical implementation with regards to informing resource allocation decisions in global health (109, 110).

## Conclusions

The choice of which type of health economic analysis to use is a matter for the decision-maker(s) and will depend on the local context, including the values and interests of the stakeholders, the question being addressed, and the resources that are being considered for reallocation (18, 111). However, in the context of resource allocation within the healthcare sector, it is important that full economic evaluations are used (which explicitly compare the costs and consequences of the interventions or health policies in question with a comparator scenario). Analyses that do not do this, even if they are incorrectly referred to as a full economic evaluation, may ignore comparisons to relevant policy alternatives and can generate misleading conclusions to policymakers.

Among the different types of full economic evaluations, cost-effectiveness/cost-utility analyses and cost-benefit analyses are typically used in this area. Although they may appear similar, it is important to recognise when interpreting these studies, that the welfarist foundation of cost-benefit analyses and the extra-welfarist foundation of cost-effectiveness/cost-utility analyses represent two fundamentally different ways of evaluating and valuing the benefits of health interventions (Box 1). There are also differences in how they consider the efficiency of resource allocation (Table 2).

Our key messages are summarised in Box 5 and Table 3. Moving forward there needs to be greater awareness within the public health field of the foundations, advantages and limitations of the different types of economic evaluations used for informing resource allocation decisions.

Box 5 **Key messages**

- In the context of resource allocation within the healthcare sector, it is important that full economic evaluations are used (which explicitly compare the costs and consequences of the interventions or health policies in question with a comparator scenario).
- Moving forward there needs to be greater awareness within the global health field of the foundations, advantages and limitations of the different types of economic evaluations used for informing resource allocation decisions (Table 3).
- Cost-effectiveness/cost-utility analyses have key advantages for directly comparing different health interventions when considering resource allocation within the healthcare sector. However, it is important to acknowledge that notable non-health benefits of interventions may not be captured and it is also difficult to compare the results of cost-effectiveness/cost-utility analyses to other sectors, limiting cross-sector comparisons.
- Cost-benefit analyses facilitate cross-sectoral comparisons and can include a wider range of benefits. However, there needs to be greater awareness of the current methods and variations in the approaches being used when placing a monetary value on health benefits (Boxes 2, 3), and the potential for the results to be misinterpreted.
- Importantly, the economic benefits being reported within such studies are typically an approximation - they are a way of summarising the health benefits experienced by a population monetarily in terms of social welfare or potential economic benefits related to productivity gains. These estimated economic benefits should not be misinterpreted as actual realisable or fiscal monetary benefits to payers or to society.

## Author Contributions

HT and YT conceived the manuscript. HT wrote the original draft. RA, LD, WI, KC, MJ, and YT contributed to the writing, reviewing, and editing of the draft. All authors read and approved the final draft.

## Author Disclaimer

The findings, interpretations, and conclusions expressed in this article do not necessarily reflect the views of the funding agencies.

## Conflict of Interest

The authors declare that the research was conducted in the absence of any commercial or financial relationships that could be construed as a potential conflict of interest.

## Publisher's Note

All claims expressed in this article are solely those of the authors and do not necessarily represent those of their affiliated organizations, or those of the publisher, the editors and the reviewers. Any product that may be evaluated in this article, or claim that may be made by its manufacturer, is not guaranteed or endorsed by the publisher.

## References

[1] Chisholm D B Evans D. Economic evaluation in health: saving money or improving care? J Med Econ. (2007) 10:325–37. 10.3111/13696990701605235

[2] Simoens S. Health economic assessment: a methodological primer. Int J Environ Res Public Health. (2009) 6:2950–66. 10.3390/ijerph6122950 20049237 PMC2800325

[3] Eddama O Coast J. A systematic review of the use of economic evaluation in local decision-making. Health Policy. (2008) 86:129–41. 10.1016/j.healthpol.2007.11.010 18192059

[4] Hayati R Bastani P Kabir MJ Kavosi Z Sobhani G. Scoping literature review on the basic health benefit package and its determinant criteria. Global Health. (2018) 14:26. 10.1186/s12992-018-0345-x 29499708 PMC5833148

[5] Luz A Santatiwongchai B Pattanaphesaj J Teerawattananon Y. Identifying priority technical and context-specific issues in improving the conduct, reporting and use of health economic evaluation in low- and middle-income countries. Health Res Policy Syst. (2018) 16:4. 10.1186/s12961-018-0280-6 29402314 PMC5800077

[6] Goodacre S McCabe C. An introduction to economic evaluation. Emerg Med J. (2002) 19:198. 10.1136/emj.19.3.198 11971826 PMC1725884

[7] Drummond M Stoddart GL Torrance GW O'Brien B. Methods for the Economic Evaluation of Health Care Programmes. 2nd edn. Oxford: Oxford Medical Publications (1997).

[8] Jit M Choi YH Edmunds WJ. Economic evaluation of human papillomavirus vaccination in the United Kingdom. BMJ. (2008) 337:a769. 10.1136/bmj.a769 18640957 PMC2500202

[9] Zarnke KB Levine MA O'Brien BJ. Cost-benefit analyses in the health-care literature: don't judge a study by its label. J Clin Epidemiol. (1997) 50:813–22. 10.1016/S0895-4356(97)00064-4 9253393

[10] Drummond MF Sculpher MJ Claxton K Stoddart GL Torrance GW. Methods for the Economic Evaluation of Health Care Programmes. 4th ed. Oxford: Oxford University Press (2015).

[11] Pitt C Goodman C Hanson K. Economic evaluation in global perspective: a bibliometric analysis of the recent literature. Health Econ. (2016) 25 (Suppl 1):9–28. 10.1002/hec.3305 26804359 PMC5042080

[12] Udeh BL. Economic evaluation studies. CHEST. (2020) 158:S88–S96. 10.1016/j.chest.2020.03.008 32658657

[13] Briggs AH O'Brien BJ. The death of cost-minimization analysis? Health Econ. (2001) 10:179–84. 10.1002/hec.584 11252048

[14] World Health Organization. Methodological approaches for cost–effectiveness and cost–utility analysis of injury prevention measures. Available online at: https://www.euro.who.int/__data/assets/pdf_file/0007/144196/e95096.pdf (accessed June 6, 2021).

[15] Sassi F. Calculating QALYs, comparing QALY and DALY calculations. Health Policy Plann. (2006) 21:402–8. 10.1093/heapol/czl018 16877455

[16] World Health Organization. WHO Guide on Standardization of Economic Evaluations of Immunization Programmes (2019). Available online at: https://www.who.int/immunization/documents/who_ivb_19.10/en/.

[17] Chang AY Horton S Jamison DT. Chapter 9. Benefit-cost analysis in disease control priorities. In: Jamison DT Gelband H Horton S Jha P Laxminarayan R Mock CN Nugent R editors. Disease Control Priorities: Improving Health and Reducing Poverty. 3rd ed. Washington, DC: The International Bank for Reconstruction and Development/The World Bank (2017). 30212058

[18] Robinson LA Hammitt JK Jamison DT Walker DG. Conducting benefit-cost analysis in low- and middle-income countries: introduction to the special issue. J Benefit-Cost Anal. (2019) 10:1–14. 10.1017/bca.2019.4 33282627 PMC7672367

[19] Thiboonboon K Kulpeng W Teerawattananon Y. An economic analysis of chromosome testing in couples with children who have structural chromosome abnormalities. PLoS ONE. (2018) 13:e0199318-e. 10.1371/journal.pone.0199318 29920550 PMC6007916

[20] Ochalek J Revill P Drummond M. Allocating Scarce Resources—Tools for Priority Setting. Global Health Economics. World Scientific Series in Global Health Economics and Public Policy. vol 5. World Scientific (2018). p. 53–73. 10.1142/9789813272378_0002

[21] Sculpher M Claxton K. Real economics needs to reflect real decisions: a response to Johnson. PharmEcon. (2012) 30:133–6. 10.2165/11596660-000000000-00000 22185184

[22] Seixas BV. Welfarism and extra-welfarism: a critical overview. Cad Saude Publica. (2017) 33:e00014317. 10.1590/0102-311x00014317 28832769

[23] Brouwer WB Culyer AJ van Exel NJ Rutten FF. Welfarism vs. extra-welfarism. J Health Econ. (2008) 27:325–38. 10.1016/j.jhealeco.2007.07.003 18179835

[24] Coast J Smith RD Lorgelly P. Welfarism, extra-welfarism and capability: the spread of ideas in health economics. Soc Sci Med. (2008) 67:1190–8. 10.1016/j.socscimed.2008.06.027 18657346

[25] Kaldor N. Welfare propositions of economics and interpersonal comparisons of utility. Econ J. (1939) 49:549–52. 10.2307/2224835

[26] Sassi F Archard L Le Grand J. Equity and the economic evaluation of healthcare. Health Technol Assess. (2001) 5:1–138. 10.3310/hta5030 11207451

[27] Hauck K Smith P Goddard M. H N P the economics of priority setting for health care: a literature review. HNP discussion paper. (2003). Available online at: https://openknowledge.worldbank.org/handle/10986/13700 (accessed June 6, 2021).

[28] Culyer AJ. The normative economics of health care finance and provision. Oxford Rev Econ Policy. (1989) 5:34–58. 10.1093/oxrep/5.1.34

[29] Edwards RT Charles JM Lloyd-Williams H. Public health economics: a systematic review of guidance for the economic evaluation of public health interventions and discussion of key methodological issues. BMC Public Health. (2013) 13:1001. 10.1186/1471-2458-13-1001 24153037 PMC4015185

[30] Bilinski A Neumann P Cohen J Thorat T McDaniel K Salomon JA. When cost-effective interventions are unaffordable: Integrating cost-effectiveness and budget impact in priority setting for global health programs. PLoS Med. (2017) 14:e1002397-e. 10.1371/journal.pmed.1002397 28968399 PMC5624570

[31] Robinson R. Cost-benefit analysis. BMJ. (1993) 307:924–6. 10.1136/bmj.307.6909.924 8241859 PMC1679054

[32] Rabarison KM Bish CL Massoudi MS Giles WH. Economic evaluation enhances public health decision making. Front Public Health. (2015) 3:164. 10.3389/fpubh.2015.00164 26157792 PMC4478374

[33] Robinson LA Hammitt JK Cecchini M Chalkidou K Claxton K Cropper M. Reference Case Guidelines for Benefit-Cost Analysis in Global Health and Development. Cambridge, MA: Harvard University (2019).

[34] Robinson LA Hammitt JK Adler M. Assessing the Distribution of Impacts in Global Benefit-Cost Analysis. Guidelines for Benefit-Cost Analysis Project, Working Paper 3 (2018). https://cdn2.sph.harvard.edu/wp-content/uploads/sites/94/2017/01/Robinson-Hammitt-Adler-Distribution-2018.03.07.pdf

[35] McIntosh E Luengo-Fernandez R. Economic evaluation. Part 1: Introduction to the concepts of economic evaluation in health care. J Family Plann Reprod Health Care. (2006) 32:107–12. 10.1783/147118906776276549 16824302

[36] Robinson LA Hammitt JK O'Keeffe L. Valuing mortality risk reductions in global benefit-cost analysis. J Benefit-Cost Anal. (2019) 10:15–50. 10.1017/bca.2018.26 32968616 PMC7473065

[37] Kanya L Sanghera S Lewin A Fox-Rushby J. The criterion validity of willingness to pay methods: a systematic review and meta-analysis of the evidence. Soc Sci Med. (2019) 232:238–61. 10.1016/j.socscimed.2019.04.015 31108330

[38] Olsen JA. Aiding priority setting in health care: is there a role for the contingent valuation method? Health Econ. (1997) 6:603–12. 9466142 10.1002/(sici)1099-1050(199711)6:6<603::aid-hec285>3.0.co;2-2

[39] Laxminarayan R Jamison DT Krupnick AJ Norheim OF. Valuing vaccines using value of statistical life measures. Vaccine. (2014) 32:5065–70. 10.1016/j.vaccine.2014.07.003 25045822

[40] Donaldson C Birch S Gafni A. The distribution problem in economic evaluation: income and the valuation of costs and consequences of health care programmes. Health Econ. (2002) 11:55–70. 10.1002/hec.642 11788982

[41] Gold MR Siegel JE Russell LB Weinstein MC. Cost-Effectiveness in Health and Medicine. New York, NY: Oxford University Press (1996).

[42] Cookson R. Willingness to pay methods in health care: a sceptical view. Health Econ. (2003) 12:891–4. 10.1002/hec.847 14601152

[43] Sen AK. Rational fools: A critique of the behavioral foundations of economic theory. Philos Public Aff. (1977) 6:317–44.

[44] Slovic P. The construction of preference. Am Psychol. (1995) 50:364. 10.1037/0003-066X.50.5.364

[45] Colmer J. What is the meaning of (statistical) life? Benefit–cost analysis in the time of COVID-19. Oxford Rev Econ Policy. (2020) 36:graa022. 10.1093/oxrep/graa022 PMC7499700 40504209

[46] Pritchard CaSM. Productivity Costs: Principles and Practice in Economic Evaluation. London: Office of Health Economics (2000).

[47] Krol M Brouwer W. How to estimate productivity costs in economic evaluations. PharmEcon. (2014) 32:335–44. 10.1007/s40273-014-0132-3 24504850

[48] Krol M Brouwer W Rutten F. Productivity costs in economic evaluations: past, present, future. PharmEcon. (2013) 31:537–49. 10.1007/s40273-013-0056-3 23620213

[49] Hung TM Shepard DS Bettis AA Nguyen HA McBride A Clapham HE. Productivity costs from a dengue episode in Asia: a systematic literature review. BMC Infect Dis. (2020) 20:393. 10.1186/s12879-020-05109-0 32493234 PMC7268537

[50] Krol M Brouwer W. Unpaid work in health economic evaluations. Soc Sci Med. (2015) 144:127–37. 10.1016/j.socscimed.2015.09.008 26421997

[51] Park M Jit M Wu JT. Cost-benefit analysis of vaccination: a comparative analysis of eight approaches for valuing changes to mortality and morbidity risks. BMC Med. (2018) 16:139. 10.1186/s12916-018-1130-7 30180901 PMC6123970

[52] Mishan EJ. Evaluation of life and limb: a theoretical approach. J Polit Econ. (1971) 79:687–705. 10.1086/259784

[53] Miller P. An Introduction to Health Economic Evaluation: The NIHR RDS for the East Midlands/Yorkshire & the Humber. (2009). Available online at: https://www.rds-yh.nihr.ac.uk/wp-content/uploads/2013/05/4_Health-Economic-Evaluation-FINAL-2009.pdf (accessed June 6, 2021).

[54] Akobundu E Ju J Blatt L Mullins CD. Cost-of-illness studies: a review of current methods. PharmEcon. (2006) 24:869–90. 10.2165/00019053-200624090-00005 16942122

[55] AJ ÓC Hanly P Skally M O'Neill C Fitzpatrick P Kapur K. Cost comparisons and methodological heterogeneity in cost-of-illness studies: the example of colorectal cancer. Med Care. (2013) 51:339–50. 10.1097/MLR.0b013e3182726c13 23358383

[56] Masters R Anwar E Collins B Cookson R Capewell S. Return on investment of public health interventions: a systematic review. J Epidemiol Commun Health. (2017) 71:827–34. 10.1136/jech-2016-208141 28356325 PMC5537512

[57] Culyer AJ Evans RG. Mark Pauly on welfare economics: Normative rabbits from positive hats. J Health Econ. (1996) 15:243–51. 10.1016/0167-6296(95)00040-2 10159112

[58] Pinkerton SD Johnson-Masotti AP Derse A Layde PM. Ethical issues in cost-effectiveness analysis. Eval Program Plann. (2002) 25:71–83. 10.1016/S0149-7189(01)00050-7

[59] Emery DD Schneiderman LJ. Cost-effectiveness analysis in health care. Hastings Cent Rep. (1989) 19:8–13. 10.2307/3562293 2501235

[60] Loomis JB. Incorporating distributional issues into benefit cost analysis: why, how, and two empirical examples using non-market valuation. J Benefit-Cost Anal. (2011) 2:1–24. 10.2202/2152-2812.1044

[61] Neumann PJ Anderson JE Panzer AD Pope EF D'Cruz BN Kim DD. Comparing the cost-per-QALYs gained and cost-per-DALYs averted literatures. Gates Open Res. (2018) 2:5. 10.12688/gatesopenres.12786.2 29431169 PMC5801595

[62] Isaranuwatchai W Archer R Teerawattananon Y Culyer A. Non-Communicable Disease Prevention: Best Buys, Wasted Buys and Contestable Buys. Open Book Publishers (2019). 10.11647/OBP.0195 PMC7190374 31992592

[63] Shillcutt SD Walker DG Goodman CA Mills AJ. Cost-effectiveness in low- and middle-income countries: a review of the debates surrounding decision rules. PharmEcon. (2009) 27:903–17. 10.2165/10899580-000000000-00000 19888791 PMC2810517

[64] Paulden M. Calculating and interpreting ICERs and net benefit. PharmEcon. (2020) 38:785–807. 10.1007/s40273-020-00914-6 32390067

[65] Verguet S Kim JJ Jamison DT. Extended cost-effectiveness analysis for health policy assessment: a tutorial. PharmEcon. (2016) 34:913–23. 10.1007/s40273-016-0414-z 27374172 PMC4980400

[66] Asaria M Griffin S Cookson R. Distributional cost-effectiveness analysis: a tutorial. Med Decis Making. (2016) 36:8–19. 10.1177/0272989X15583266 25908564 PMC4853814

[67] Cookson R Griffin S Culyer AJ Norheim OF. Distributional Cost-Effectiveness Analysis: Quantifying Health Equity Impacts and Trade-Offs. Oxford University Press (2020).

[68] World Health Organization. Constitution. Available online at: https://www.who.int/about/governance/constitution (accessed June 6, 2021).

[69] Priorities in Health. In: Jamison DT Breman JG Measham AR Alleyne G Claeson M Evans DB editors. Washington, DC: The International Bank for Reconstruction and Development/The World Bank (2006). 21089239

[70] GEAR. Guidelines Comparison. Available online at: http://www.gear4health.com/gear/health-economic-evaluation-guidelines (accessed June 6, 2021).

[71] Husereau D Drummond M Petrou S Carswell C Moher D Greenberg D. Consolidated health economic evaluation reporting standards (CHEERS) statement. BMJ. (2013) 346:f1049. 10.1136/bmj.f1049 23529982

[72] World Health Organization. Making choice in health: WHO Guide to Cost Effective Analysis. Geneva: WHO (2003).

[73] iDSI Reference Case for Economic Evaluation. Available online at: https://www.idsihealth.org/resource-items/idsi-reference-case-for-economic-evaluation/ (accessed June 6, 2021).

[74] Neumann PJ Thorat T Zhong Y Anderson J Farquhar M Salem M. A Systematic review of cost-effectiveness studies reporting cost-per-DALY averted. PLoS ONE. (2016) 11:e0168512. 10.1371/journal.pone.0168512 28005986 PMC5179084

[75] Neumann PJ. Costing and perspective in published cost-effectiveness analysis. Med Care. (2009) 47(7 Suppl 1):S28–32. 10.1097/MLR.0b013e31819bc09d 19536023

[76] King CH Bertino AM. Asymmetries of poverty: why global burden of disease valuations underestimate the burden of neglected tropical diseases. PLoS Neglected Trop Dis. (2008) 2:e209. 10.1371/journal.pntd.0000209 18365036 PMC2267491

[77] King CH. Health metrics for helminth infections. Acta Trop. (2015) 141 (Pt B):150–60. 10.1016/j.actatropica.2013.12.001 24333545 PMC4055550

[78] Nord E. Disability weights in the Global Burden of Disease 2010: unclear meaning and overstatement of international agreement. Health Policy. (2013) 111:99–104. 10.1016/j.healthpol.2013.03.019 23608637

[79] World Health Organization. WHO methods and data sources for global causes of death 2000–2011. (2013). Available online at: https://www.who.int/healthinfo/global_burden_disease/GlobalCOD_method_2000-2011.pdf (accessed June 6, 2021).

[80] King NB Harper S Young M Berry SC Voigt K. The impact of social and psychological consequences of disease on judgments of disease severity: an experimental study. PLoS One. (2018) 13(4):e0195338. 10.1371/journal.pone.0195338 29664972 PMC5903632

[81] Hausman DM. Health, well-being, and measuring the burden of disease. Popul Health Metrics. (2012) 10:13. 10.1186/1478-7954-10-13 22852827 PMC3487868

[82] Voigt K King NB. Disability weights in the global burden of disease 2010 study: two steps forward, one step back? Bull World Health Org. (2014) 92:226–8. 10.2471/BLT.13.126227 24700983 PMC3949595

[83] Hung TM Clapham HE Bettis AA Cuong HQ Thwaites GE Wills BA. the estimates of the health and economic burden of dengue in Vietnam. Trends Parasitol. (2018) 34:904–18. 10.1016/j.pt.2018.07.007 30100203 PMC6192036

[84] Whitehead SJ Ali S. Health outcomes in economic evaluation: the QALY and utilities. Br Med Bull. (2010) 96:5–21. 10.1093/bmb/ldq033 21037243

[85] McDonough CM Tosteson AN. Measuring preferences for cost-utility analysis: how choice of method may influence decision-making. PharmEcon. (2007) 25:93–106. 10.2165/00019053-200725020-00003 17249853 PMC3046553

[86] Pettitt DSR. The limitations of QALY: a literature review. J Stem Cell Res Ther. (2016) 6:4. 10.4172/2157-7633.1000334

[87] Stamuli E. Health outcomes in economic evaluation: Who should value health? Br Med Bull. (2011) 97:197–210. 10.1093/bmb/ldr001 21285110

[88] Teerawattananon Y Luz AC Culyer A Chalkidou K. Charging for the use of survey instruments on population health: the case of quality-adjusted life years. Bull World Health Organ. (2020) 98:59–65. 10.2471/BLT.19.233239 31902963 PMC6933437

[89] Schwarzer R Rochau U Saverno K Jahn B Bornschein B Muehlberger N. Systematic overview of cost–effectiveness thresholds in ten countries across four continents. J Comp Eff Res. (2015) 4:485–504. 10.2217/cer.15.38 26490020

[90] Thokala P Ochalek J Leech AA Tong T. Cost-effectiveness thresholds: the past, the present and the future. PharmEcon. (2018) 36:509–22. 10.1007/s40273-017-0606-1 29427072

[91] WHO Commission on Macroeconomics Health World Health Organization. Macroeconomics and Health: Investing in Health for Economic Development: Executive Summary/Report of the Commission on Macroeconomics and Health. Geneva: World Health Organization (2001).

[92] Newall AT Jit M Hutubessy R. Are current cost-effectiveness thresholds for low- and middle-income countries useful? Examples from the world of vaccines. PharmEcon. (2014) 32:525–31. 10.1007/s40273-014-0162-x 24791735

[93] Marseille E Larson B Kazi DS Kahn JG Rosen S. Thresholds for the cost-effectiveness of interventions: alternative approaches. Bull World Health Org. (2015) 93:118–24. 10.2471/BLT.14.138206 25883405 PMC4339959

[94] Woods B Revill P Sculpher M Claxton K. Country-level cost-effectiveness thresholds: initial estimates and the need for further research. Value Health. (2016) 19:929–35. 10.1016/j.jval.2016.02.017 27987642 PMC5193154

[95] Leech AA Kim DD Cohen JT Neumann PJ. Use and misuse of cost-effectiveness analysis thresholds in low- and middle-income countries: trends in cost-per-DALY studies. Value Health. (2018) 21:759–761. 10.1016/j.jval.2017.12.016 30005746 PMC6041503

[96] Bertram MY Lauer JA De Joncheere K Edejer T Hutubessy R Kieny M-P. Cost-effectiveness thresholds: pros and cons. Bull World Health Org. (2016) 94:925–30. 10.2471/BLT.15.164418 27994285 PMC5153921

[97] GEAR. It is difficult to interpret and use the results of the cost effectiveness analysis (CEA). Available from: http://www.gear4health.com/gear/mind-map/11 (accessed June 6, 2021).

[98] Culyer AJ. Cost-effectiveness thresholds in health care: a bookshelf guide to their meaning and use. Health Econ Policy Law. (2016) 11:415–32. 10.1017/S1744133116000049 26906561

[99] Revill P Ochalek J Lomas J Nakamura R Woods B Rollinger A. Cost-Effectiveness Thresholds: Guiding Health Care Spending for Population Health Improvement. Global Health Economics. World Scientific Series in Global Health Economics and Public Policy. Vol 5. World Scientific (2018). p. 75–97. 10.1142/9789813272378_0003

[100] Weatherly H Drummond M Claxton K Cookson R Ferguson B Godfrey C. Methods for assessing the cost-effectiveness of public health interventions: key challenges and recommendations. Health Policy. (2009) 93:85–92. 10.1016/j.healthpol.2009.07.012 19709773

[101] Jit M Hutubessy R Png ME Sundaram N Audimulam J Salim S. The broader economic impact of vaccination: reviewing and appraising the strength of evidence. BMC Med. (2015) 13:209. 10.1186/s12916-015-0446-9 26335923 PMC4558933

[102] Payne K McAllister M Davies LM. Valuing the economic benefits of complex interventions: when maximising health is not sufficient. Health Econ. (2013) 22:258–71. 10.1002/hec.2795 22308053

[103] Brouwer WB Koopmanschap MA Rutten FF. Productivity costs measurement through quality of life? A response to the recommendation of the Washington Panel. Health Econ. (1997) 6:253–9. 9226143 10.1002/(sici)1099-1050(199705)6:3<253::aid-hec266>3.0.co;2-6

[104] Brouwer WB Koopmanschap MA Rutten FF. Productivity costs in cost-effectiveness analysis: numerator or denominator: a further discussion. Health Econ. (1997) 6:511–4. 9353652 10.1002/(sici)1099-1050(199709)6:5<511::aid-hec297>3.0.co;2-k

[105] Sculpher M. The role and estimation of productivity costs in economic evaluation. In: Michael MD Alistair McGuire editors. Economic Evaluation in Health Care: Merging Theory With Practice. Oxford: Oxford University Press (2001). p. 94–112.

[106] Olsen JA Richardson J. Production gains from health care: what should be included in cost-effectiveness analyses? Soc Sci Med. (1999) 49:17–26. 10.1016/S0277-9536(99)00116-1 10414837

[107] Liljas B. How to calculate indirect costs in economic evaluations. PharmEcon. (1998) 13:1–7. 10.2165/00019053-199813010-00001 10175982

[108] Brock D. Ethical issues in the use of cost effectiveness analysis for the prioritization of health resources. In: Khushf G editor. Handbook of Bioethics: Taking Stock of the Field from a Philosophical Perspective. Dordrecht: Springer Netherlands (2004). p. 353–80.

[109] Baltussen R Marsh K Thokala P Diaby V Castro H Cleemput I. Multicriteria decision analysis to support health technology assessment agencies: benefits, limitations, and the way forward. Value Health. (2019) 22:1283–8. 10.1016/j.jval.2019.06.014 31708065

[110] Cookson R Mirelman AJ Griffin S Asaria M Dawkins B Norheim OF. Using cost-effectiveness analysis to address health equity concerns. Value Health. (2017) 20:206–12. 10.1016/j.jval.2016.11.027 28237196 PMC5340318

[111] Culyer AJ Chalkidou K. Economic evaluation for health investments en route to universal health coverage: cost-benefit analysis or cost-effectiveness analysis? Value Health. (2019) 22:99–103. 10.1016/j.jval.2018.06.005 30661640 PMC6347566

